# The Nurses' Co-Operation

**Published:** 1920-12-04

**Authors:** Marie Eglinton Murray, Sarah E. Stephenson, Geraldine Bremner, Mary E. Elliott

**Affiliations:** Nurses' Representatives on the Committee of Management. The Nurses' Co-operation, 22 Langham Street, London, W. 1; Nurses' Representatives on the Committee of Management. The Nurses' Co-operation, 22 Langham Street, London, W. 1; Nurses' Representatives on the Committee of Management. The Nurses' Co-operation, 22 Langham Street, London, W. 1; Nurses' Representatives on the Committee of Management. The Nurses' Co-operation, 22 Langham Street, London, W. 1


### The Nurses' Co-operation.

*To the Editor of* The Hospital.

Sir,—We shall be grateful if you will grant to us, the undersigned representatives of the nurses on the staff of the Nurses' Co-operation, the hospitality of your columns to assist us in removing an erroneous impression which may have been created by certain references to that institution made in the course of recent litigation to which the Co-operation was not a party.

The Nurses' Co-operation is a company limited by guarantee, now composed of about thirty members. Its principal objects are to maintain an office or agency where nurses may be engaged, and a home for nurses. It also bears the whole expense of an accident insurance scheme and contributes towards a Sickness Fund for the benefit of the nurses. Its constitution embodies three fundamental rules: (1) Its income and property must be devoted solely towards the promotion of its objects; (2) no member is permitted to participate in profits or assets; and (3) if the Co-operation is dissolved its property must be transferred to some other institution *having similar objects.* Nurses who can be engaged through the agency of the Co-operation are spoken of as being "on the staff." Their number is now about 470. They are *not* members of the Co-operation, owing to the rule that no member may derive any profit. These nurses contribute to the funds of the Co-operation a percentage of the fees paid to them by their patients—5 per cent. in some cases and 7½ per cent. in others.

The Co-operation is managed by a Committee of seventeen, of whom ten are elected by the nurses. At the present time nine of these ten nurses' representatives are working nurses, the tenth being a member who was formerly a nurse. The accumulated funds are represented by the following assets: The Howard de Walden Nurses' Home and Club (appropriated for the use of the nurses on the staff) and the Co-operation's offices at 22 Langham Street, with their furniture and fittings, the book value being £20,000; cash in hand, investments, and shares, worth about £3,000 These assets have been acquired by means of donations (including £7,500 from the late Lady Howard de Walden), members' subscriptions, and accumulations of surplus income when there has been a surplus—in some years current expenditure on the objects of the institution has exceeded the income.

The public may rest assured that the nurses who may be engaged through this Co-operation have the full benefit of the institution's assets and income, and of the fees which they earn. References have been made to salaries of £30 and £40 a year and working days of sixteen hours. These cannot apply to the nurses attached to this institution, whose rules lay down that the minimum fee is £3 3s. a week, while bed and board and cost of laundry and travelling have to be found by the patient, and the hours of duty are limited to twelve, *inclusive* of time for outdoor exercise.—We are, yours faithfully,

Marie Eglinton Murray,
Sarah E. Stephenson,
Geraldine Bremner,
Mary E. Elliott,
Nurses' Representatives on the Committee of Management.

The Nurses' Co-operation, 22 Langham Street, London, W. 1, November 26, 1920.

P.S.—We may add that this letter is sent by the authority of all the nine nurses' representatives.

